# Postnatal periodontal ligament as a novel adult stem cell source for regenerative corneal cell therapy

**DOI:** 10.1111/jcmm.13589

**Published:** 2018-03-13

**Authors:** Gary Hin‐Fai Yam, Ericia Pei‐Wen Teo, Melina Setiawan, Matthew J Lovatt, Nur Zahirah Binte M Yusoff, Matthias Fuest, Bee‐Tin Goh, Jodhbir S Mehta

**Affiliations:** ^1^ Tissue Engineering and Stem Cell Group Singapore Eye Research Institute Singapore; ^2^ Ophthalmology and Visual Science Academic Clinical Research Program Duke‐National University of Singapore Graduate Medical School Singapore; ^3^ Department of Ophthalmology RWTH Aachen University Aachen Germany; ^4^ Department of Oral and Maxillofacial Surgery National Dental Centre Singapore; ^5^ Cornea and External Eye Disease Service Team Singapore National Eye Centre Singapore; ^6^ School of Material Science and Engineering Nanyang Technological University Singapore

**Keywords:** cornea, corneal stromal keratocytes, differentiation, periodontal ligament, stem cells

## Abstract

Corneal opacities are a leading cause of global blindness. They are conventionally treated by the transplantation of donor corneal tissue, which is, restricted by a worldwide donor material shortage and allograft rejection. Autologous adult stem cells with a potential to differentiate into corneal stromal keratocytes (CSKs) could offer a suitable choice of cells for regenerative cell therapy. Postnatal periodontal ligament (PDL) contains a population of adult stem cells, which has a similar embryological origin as CSK, that is cranial neural crest. We harvested PDL cells from young adult teeth extracted because of non‐functional or orthodontic reason and differentiated them towards CSK phenotype using a two‐step protocol with spheroid formation followed by growth factor and cytokine induction in a stromal environment (human amnion stroma and porcine corneal stroma). Our results showed that the PDL‐differentiated CSK‐like cells expressed CSK markers (CD34, ALDH3A1, keratocan, lumican, CHST6, B3GNT7 and Col8A2) and had minimal expression of genes related to fibrosis and other lineages (vasculogenesis, adipogenesis, myogenesis, epitheliogenesis, neurogenesis and hematogenesis). Introduction of PDL spheroids into the stroma of porcine corneas resulted in extensive migration of cells inside the host stroma after 14‐day organ culture. Their quiescent nature and uniform cell distribution resembled to that of mature CSKs inside the native stroma. Our results demonstrated the potential translation of PDL cells for regenerative corneal cell therapy for corneal opacities.

## 1 INTRODUCTION

The cornea provides a physical and biological barrier to protect inner eye tissues and its transparency allows efficient light transmission and refraction for normal vision and visual acuity. The corneal stroma contains collagen fibrils (mainly type I) aligned in the form of lamellae, which run in orthogonal orientations to provide the corneal strength and transparency.^1^ Corneal stromal keratocytes (CSKs) located between collagen lamellae synthesize and deposit collagens and keratan sulphate proteoglycans (KSPGs; lumican, keratocan and mimecan) that regulate collagen fibril alignment and interfibrillar spacing, which are crucial for stromal matrix organization, corneal mechanics and transparency.^2^ Developmentally, CSKs are derived from the ocular mesenchyme of cranial neural crest (NC) origin.^3^, ^4^ In adult corneas, they remain quiescent and extend dendritic cell processes to neighbouring keratocytes forming a highly organized syncytium. Besides collagens and KSPGs, they also express stromal crystallins, such as aldehyde dehydrogenases (ALDH, type 1A1 and 3A1), α‐enolase, lactic dehydrogenase and transketolase, which match the refractive indices between inter‐ and extra‐cellular regions, contributing to transparency.^5^ Trauma to the corneal stroma or infectious keratitis will activate CSKs to transform into stromal fibroblasts (SFs) with a loss of keratocyte features. SFs are proliferative and acquire a different set of tissue healing‐related genes compare to CSK's, for example fibronectin, α5‐integrin and/or α‐smooth muscle actin (αSMA) (when SFs transit to myofibroblasts during scar formation).^6^ SFs mediate extracellular matrix (ECM) contraction and disrupt the organized lamellar alignment, causing diminished transparency.

“Corneal opacities” are a leading cause of worldwide blindness and are estimated to affect over 10 millions of people (information obtained from WHO global blindness data and WHO 2002 sub‐regional causes).^7^, ^8^ Conventionally, advanced stages of opacities are treated by keratoplasty using donor corneas.^9^ However, the use of cadaveric donor tissue is limited with a worldwide shortage, and there is a risk of allograft rejection in the longer term (up to 38% 10‐year graft failure rate).^10^, ^11^ Hence, new strategies should be developed to replace the defective CSKs and to eliminate or prevent stromal scarring.^12^ Autologous cell therapy involving an adult stem cell source is an attractive option.

Many adult stem cell types are known to transdifferentiate into cell types other than the tissue they reside. Unlike induced pluripotent stem cells, the conversion of adult stem cells can be direct, efficient and bypass the pluripotent cell state, which often elicits safety concerns and risk of tumorigenesis.^13^ Many studies have reported transdifferentiation via the forced expression of transcription factors or the provision of an appropriate niche and trophic factors.^14^, ^15^, ^16^, ^17^ Although the exact mechanism is undetermined, the presence of suitable environment and enhancement of functional activity of specific niche (with signal mediators) may suffice to drive the differentiation of adult stem cells towards the desired phenotypes.

In recent years, dental stem cells have received much attention in cell regeneration research because of its easy accessibility, plasticity and applicability in regenerative medicine.^18^, ^19^ Among the known dental stem cells,^20^, ^21^ periodontal ligament‐derived stem cells (PDLSCs) and dental pulp stem cells (DPSCs) originate from the cranial NC and share similar developmental pathways as CSKs.^22^, ^23^ Isolation of PDL tissue by scraping from tooth root surface is relatively easier than that for dental pulp cells, which requires cracking the tooth and this could induce primary cell death. Besides DPSCs, the loose dental pulp tissue contains nerve and blood vessels. Tooth extraction induces lesions to the pulp nerve and endings, which cause the release of inflammatory cytokines, such as IL‐1 and TNFα, and this would impact on DPSCs’ physiological status, such as survival and multipotency.^24^, ^25^

A recent study has illustrated that DPSCs could differentiate into CSK‐like cells expressing keratocan (KERA), a unique marker for CSKs, and KSPGs.^26^ DPSC‐derived CSKs, when transplanted to mouse stroma, deposited stromal ECM and maintained the corneal clarity. PDLSCs, on the other hand, have clonal proliferation capability and express specific markers of mesenchymal stem cells (MSCs), embryonic stem cells (ESCs) and neural crest stem cells (NCSCs).^22^, ^27^, ^28^ They exhibit multi‐lineage potential under directed differentiation, including adipogenic, chondrogenic, osteogenic and neurogenic.^29^, ^30^, ^31^ Enriched connexin43‐expressing PDLSCs generated cells resembling embryonic germ layers: mesodermal, ectoderm and endoderm.^32^ There are other studies showing their transdifferentiation potential to pancreatic islet‐like cells with functional insulin production, to skeletal muscle cells with myotubular‐like structures, to vascular cells and to photoreceptor‐like cells with excitatory potential.^33^, ^34^, ^35^, ^36^

This study investigated the potential of adult PDLSCs to differentiate into CSKs through NC cell enrichment and treatment with a combination of appropriate microenvironment and growth factors. Our results have provided useful information to demonstrate PDL as a novel and autologous cell source for regenerative corneal cell therapy.

## 2 MATERIALS AND METHODS

### 2.1 Human teeth collection, PDLSC isolation and culture

Permanent teeth (including third molar) were extracted from 41 young Chinese (age range: 13‐28 years old; male‐to‐female ratio: 3:4) because of non‐functional or orthodontic reasons at National Dental Centre, Singapore. The study protocol was approved by the Centralized Institutional Research Board, SingHealth, Singapore (Ref 2013/287/A) and carried out in accordance with the tenets of the Declaration of Helsinki and informed consents were obtained. Donor subjects had good oral hygiene, and no history of smoking, previous radiotherapy in the head and neck region, periodontal diseases or other active dental infection.

The extracted teeth were delivered in Dulbecco's modified Eagle's medium (DMEM)/F12 medium (Invitrogen, Carlsbad, CA, USA) added with 300 μg/mL streptomycin sulphate, 3% amphotercin B and 3% amphopterycin B (antibiotics‐antimycotes, Invitrogen) on ice to the culture facility within 6 hours after extraction. PDL cell isolation protocol was followed as previously reported.^35^ Briefly, after extensive PBS washes to remove blood traces, PDL tissue was scraped mechanically along the middle one‐third of root surface, finely cut and digested with 100 μg/mL collagenase I (Worthingon, Lakewood, NJ, USA) in DMEM/F12 medium containing 0.5% foetal bovine serum (FBS, Invitrogen) and antibiotics for 4‐6 hours with agitation (100 rpm) at 37°C. Single cell suspension was obtained by passing through a cell strainer (40 μm pore size, Falcon, Thermo Fisher Sci), and the collected cells were cultured in DMEM/F12 supplemented with 5% FBS and antibiotics. When PDL cell culture reached about 70% confluence, it was subpassaged by trypsinization and seeded at a density of 5 × 10^4^ cells/cm^2^ in a new culture vessel. Cells at passage (P) 3‐5 were used in the experiments.

### 2.2 Cornea stromal keratocyte isolation and culture

Three research grade cadaveric corneal tissues deemed unsuitable for transplantation (Table S1) were purchased from Lions Eye Institute for Transplant and Research Inc. (Tampa, FL, USA) following institutional review board approval, in accordance with approved guidelines. Consent was taken at the time of retrieval by next of kin for use in research. Corneal tissues were transported in Optisol‐GS (Bausch&Lomb Surgical, Irvine, CA, USA) at 4°C. The central button (8 mm in diameter) was treated with dispase II (20 mg/mL, Roche, Switzerland) to remove corneal epithelium and endothelium. The remaining stroma was trimmed into tiny fragments (<0.5 mm^3^) and digested with collagenase I (1 mg/mL) for <8 hours at 37°C. Primary culture of activated keratocytes was established using low serum ERI protocol as reported earlier.^37^ At P4, activated keratocytes were kept in serum‐free ERI culture to obtain CSKs and in 5% serum culture to obtain SFs, respectively, for 7 days. After characterization by specific marker expression, these cultures were used as control cell types.^38^ Similarly, primary CSK cultures from adult monkey (n = 4) and rabbit (n = 5) were established using the same ERI protocol and P4 cells were used in the experiments.

### 2.3 Human amnion membrane preparation

Human foetal amnion (n = 1) was isolated from placenta (mother younger than 40 years old) after caesarean section, with written consent and institutional review board‐approved protocol (CIRB Ref: 2015/2607). After rinses with sterile saline to remove blood traces, the amnion (AM) was manually peeled and separated from the chorion. The AM portion distal from the placenta was collected, trimmed into 4 × 5 cm^2^ dimension, placed on sterile nitrocellulose paper (0.45 μm, Bio‐Rad, Herculus, CA, USA) with the stromal side facing up and stored in DMEM/glycerol (1:1 vol/vol) at −80°C until use.

### 2.4 Spheroid culture

Primary PDL cells were suspended at a density of 500 cells/mL in DMEM/F12 added with 1% B27 (Invitrogen), 1% N2 (Invitrogen), 0.5 μmol/L β‐mecaptoethanol (Sigma), 15% chick embryo extract (CEE, Gemini Bio‐Products, West Sacramento, CA, USA) and 5% FBS on an ultra‐low attachment culture plate (Corning, NY, USA) for up to 14 days. Spheroids with diameter >50 μm were collected for CSK fate induction.

### 2.5 Keratocyte fate induction of PDL spheroids on human amnion stroma

Periodontal ligament spheroids were disaggregated using collagenase I (2 mg/mL) incubated for 60 minutes at 37°C, followed by repeated pipetting. The cell clusters were further treated with 0.02% trypsin‐EDTA (Invitrogen) for 5‐10 minutes and passed through cell strainer (40 μm pore size) to obtain single cell suspension. After washes, cells were resuspended in serum‐free CSK induction medium, which was DMEM/F12 added with MEM amino acids (Invitrogen), MEM non‐essential amino acids (Invitrogen), insulin‐selenate‐transferin (Invitrogen), l‐ascorbate 2‐phosphate (_L_A_2_P, 1 mmol/L, Sigma), basic fibroblast growth factor (bFGF, 20 ng/mL, Invitrogen) and transforming growth factor β3 (TGFβ3, 0.1 ng/mL, Invitrogen). Both single cell and spheroid suspensions were applied to collagen I‐coated culture surface. In parallel, they were also placed on human AM stroma, of which the thawed AM was washed extensively with PBS to remove traces of glycerol and then placed on culture plate (60 mm diameter) with stromal side facing up. The cultures were maintained up to 15 days with medium replenished every 3 days.

### 2.6 Intrastromal injection of PDL spheroids into porcine corneas

Periodontal ligament spheroids (about 50 μm diameter) were labelled with Molday ION‐Evergreen™ reagent (BioPAL, Worcester, MA, USA) for 48 hours before collection. After washing twice with PBS, the spheroids were resuspended in normal saline (0.9% sodium chloride, B Braun, Singapore) at a concentration of 5 spheroids per μL. Porcine eyes (n = 34) were collected from local abattoir and disinfected by Povidone‐iodine (ICN Pharma., Costa Mesa, CA, USA) for 20 minutes followed by extensive PBS washes. The cornea with surrounding scleral rim was carefully isolated and filled with 1% low melting point agarose (Sigma) in M199 medium (Invitrogen) supplemented with antibiotics for support. Positioned with epithelial side up, four stromal tunnels with length of 5‐6 mm were created using 23G needles at a depth of about 100 μm from the corneal surface. A volume of 3‐ to 4‐μL spheroid suspension was slowly injected into the tunnel, after which the surface pore was closed by a drop of agarose solution. The corneas were maintained in CSK induction medium for up to 14 days with fresh medium replenished every 2 days.

### 2.7 Flow cytometry

Cells were fixed with neutral buffered 2% paraformaldehyde (Sigma‐Aldrich, St Louis, MO, USA) for 20 minutes. After PBS (phosphate buffered saline, 0.01 mol/L, First Base, Singapore) washes and permeabilized with 0.1% saponin (Sigma), cells were incubated with monoclonal antibodies (Table S2) followed by appropriate secondary antibody and propidium iodide (Sigma). After washes, cells were suspended in PBS (10^5^ cells/mL) and analysed by FACSVerse™ System (BD Biosciences, Singapore) with a minimum of 10 000 events in each experiment.

### 2.8 Immunofluorescence

Samples were fixed with neutral buffered 4% paraformaldehyde. After quenching with ice‐cold 50 mmol/L ammonium chloride (Sigma), samples were permeabilized with 0.15% saponin and blocking with 1% bovine serum albumin (Sigma) and 2% normal goat serum (Invitrogen), followed by incubation with primary antibodies (Table S2) for 2 hours at room temperature. After PBS washes, they were labelled with appropriate Red‐X or Alexa488‐conjugated IgG secondary antibody (Jackson ImmunoRes Lab, West Grove, PA, USA) and fluorescein‐conjugated phalloidin (Invitrogen). After washes, samples were mounted with Fluoroshield with DAPI (4′,6‐diamidino‐2‐phenylindole; Santa Cruz Biotech, Santa Cruz, CA, USA) and viewed under fluorescence microscopy (AxioImager Z1, Carl Zeiss, Oberkochen, Germany) or confocal laser‐scanning microscopy (SP8; Leica Microsystems, Wetzlar, Germany).

### 2.9 Quantitative PCR

Samples in RLT buffer (Qiagen, Valencia, CA, USA) or Trizol (Sigma) were processed for total RNA extraction using RNeasy kit (Qiagen) and on‐column RNase‐free DNase kit (Qiagen) according to manufacturer's protocol. Total RNA (1 μg) was reverse transcribed using Superscript III RT‐PCR kit (Invitrogen) with random hexanucleotide primer (10 ng/mL, Invitrogen). Gene expression was assayed with specific primer pairs (Table S3) by quantitative real‐time PCR (qPCR) using Sybr Green Supermix (Bio‐Rad) in GFX96 Real‐time System (Bio‐Rad). Experiments were run in quadruplicate. Relative gene expression of each sample (ΔCT) was normalized by the mean CT of the housekeeping gene glyceraldehyde‐3‐phosphate dehydrogenase (GAPDH) (CT_GAPDH_) and expressed as mean and standard deviation (SD).

### 2.10 Statistical analysis

Mann‐Whitney *U*‐test was used to compare the gene expression levels between treatment and control groups. Results were described as mean and SD. All statistical calculation was performed using SPSS 20.0 (SPSS, Chicago, IL, USA). *P *<* *.05 was considered statistically significant.

## 3 RESULTS

**Figure 1:**
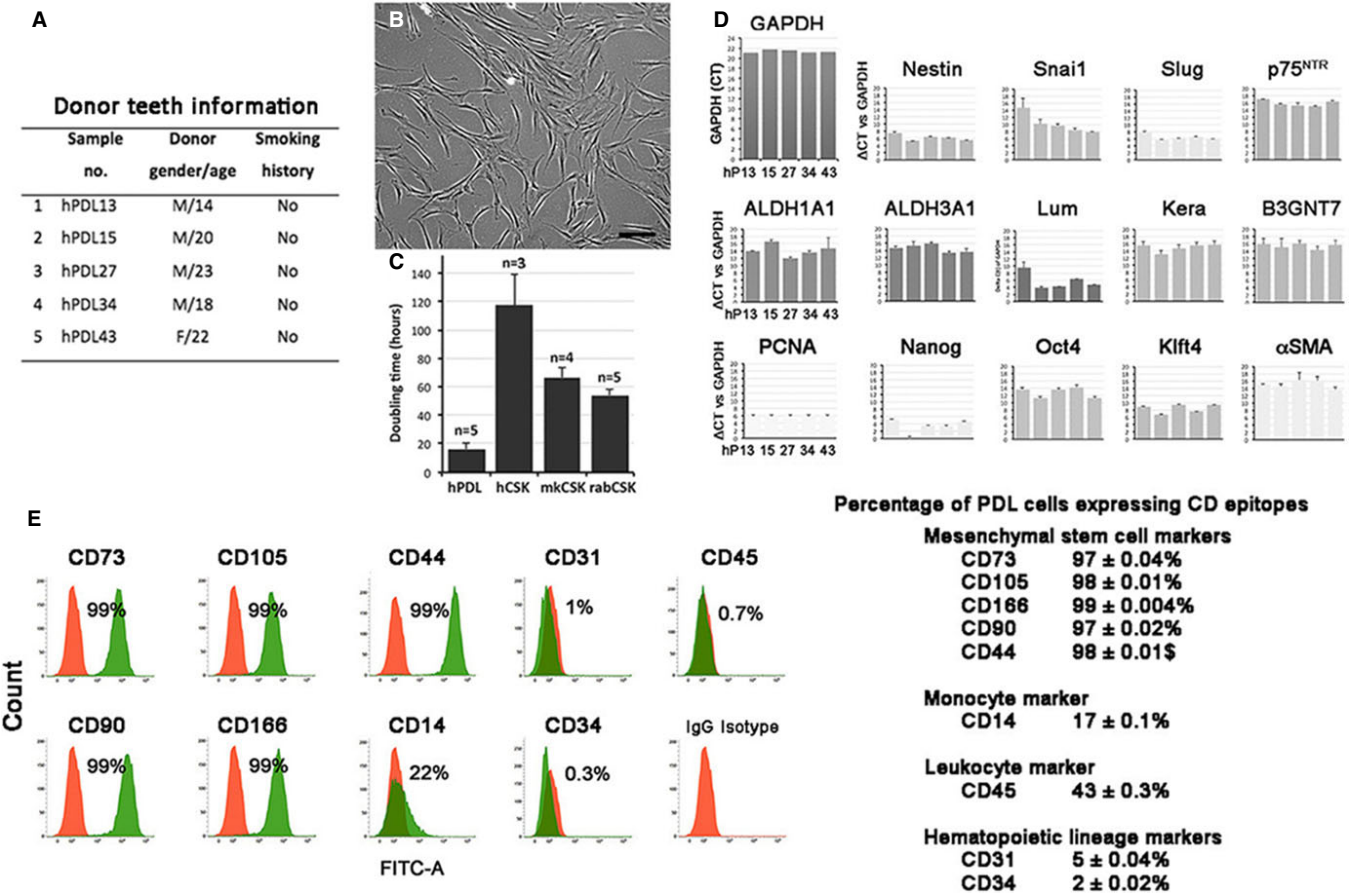
Primary periodontal ligament (PDL) cell culture and characterization. A, Donor teeth information in this study. B, A representative phase contrast image of primary hPDL15 culture at passage 3. Scale bar: 50 μm. C, Growth assessment by cell doubling time comparing between human primary PDL cells (n = 5 collections) and primary corneal stromal keratocytes (CSKs) isolated from human (n = 3), monkey (n = 4) and rabbit (n = 5). Each bar represents mean ± standard deviation (SD). D, qPCR analysis showing the expression of neural crest genes (*nestin*,* Snai1*,* Slug*,* p75*), multipotency genes (*Nanog*,* Klf4*,* Oct4*), cell proliferation nuclear antigen (*PCNA*), CSK genes (*ALDH1A1*,* ALDH3A1*,* KERA*,* LUM*,* B3GNT7*) and contractile fibroblasts (α*SMA*) in 5 primary PDL cultures. The expression was represented as ΔCT compared to housekeeping GAPDH expression. E, Flow cytometric histograms showing the event profiling of CD markers for mesenchymal stem cells, monocytes, leucocytes and hematopoietic lineage cells. Red‐coloured histograms denote the isotype control

We collected teeth from 41 Chinese individuals and successfully established 21 primary PDL cultures (success rate: 51.2%). The failed cultures were because of contamination issues or the absence of viable cells. Among those established cultures, we randomly selected five primary cultures for the CSK induction study and all of these donors were non‐smokers and had good to excellent oral hygiene status reported by dental surgeon (GBT) (Figure 1A). Each primary PDL culture was derived from PDL tissues of a single donor and we did not pool cells from different individuals. This would demonstrate the unique responsiveness of PDL cells towards CSK differentiation irrespective of individual variability.

### 3.1 PDL cell characterization

The primary cells were adherent and displayed clonal growth forming loose colonies with migratory cells at the periphery, whereas cells at central region displayed more intercellular connections via dendritic cell processes (Figure 1B). Their growth rate, assessed by the cell doubling time, was greater than that of CSKs from different species (human, monkey and rabbit) (human PDL: 17.2 ± 2.7 hours; human CSK: 116.7 ± 22.2 hours; monkey CSK: 63.8 ± 8.9 hours and rabbit CSK: 55.4 ± 3.7 hours) (Figure 1C). Cells at P3 were characterized for specific gene expression representing NC‐derived cells using qPCR. Figure 1D showed deltaCT values of various genes with reference to the housekeeping *GAPDH*. Strong expression of NC genes (*nestin*,* Snai1*,* Slug*), multipotency genes (*Nanog*,* Klf4*) and cell proliferation nuclear antigen (*PCNA*) was detected. These cells had low to negligible gene expression for multipotent stem/progenitor cell (*OCT4*,* p75* ^*NTR*^) and contractile fibroblasts (α*SMA*). By flow cytometry, more than 97% PDL cells positively expressed MSC markers (CD44, 73, 90, 105, 166). Immunofluorescence illustrated the expression of SSEA‐1 and STRO‐1 (Figure S1). However, they had much lower expression of hematopoietic lineage‐related CD31 (<5%) and CD34 (<2%), as well as CD14 (monocyte marker, 17%) and CD45 (leucocyte marker, 43%) (Figures 1E and S2, Table S4). The multipotency of human PDL cells was validated through induced differentiation along osteogenesis by Alizarin Red S (Figure S3; method in Supporting Information). Overall, our result indicated that primary PDL cells possessed the characteristics of multipotent stem/progenitor cells. We also verified the negligible expression of CSK gene markers (*ALDH1A1*,* ALDH3A1*,* KERA* and Kera‐synthesizing enzyme *B3GNT7*) in primary PDL cells; however, lumican (*LUM*) was strongly expressed (Figure 1D).

### 3.2 CSK fate commitment via PDL spheroid formation

**Figure 2:**
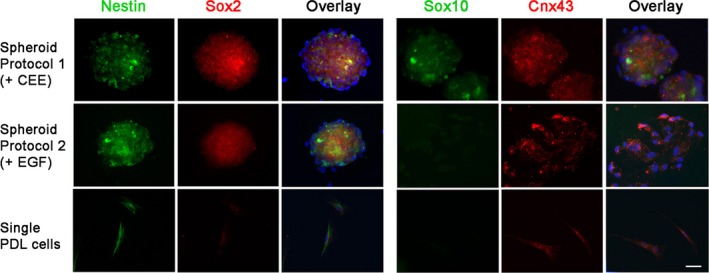
Characterization of periodontal ligament (PDL) spheroids. Spheroids generated under low attachment culture for 5 d using two protocols (with 5% chick embryo extract or with 10 ng/mL EGF) were analysed for the expression of neural crest genes (nestin, Sox2, Sox10) and connexin43, and the staining was compared to control PDL cells. Scale bar: 20 μm

A two‐step differentiation protocol involving an initial spheroid formation under low attachment culture followed by CSK induction was carried out. PDL cells at seeding density of 500 cells/mL generated spheroids with diameters up to 100 μm in 14 days of suspension culture. The spheroid formation efficiency was 4.8 ± 1.3%. Comparing between supplements, culture added with 5% CEE generated more intact spheroids than that supplemented with 20 ng/mL EGF, a protocol reported to expand palatal NCSCs.^39^ The centrally located cells of CEE‐generated PDL spheroids expressed nestin, Sox2 and Sox10, suggesting the NC identity (Figure 2). However, Sox2 and Sox10 were negligibly expressed in EGF‐generated spheroids as well as in single PDL cells.

**Figure 3:**
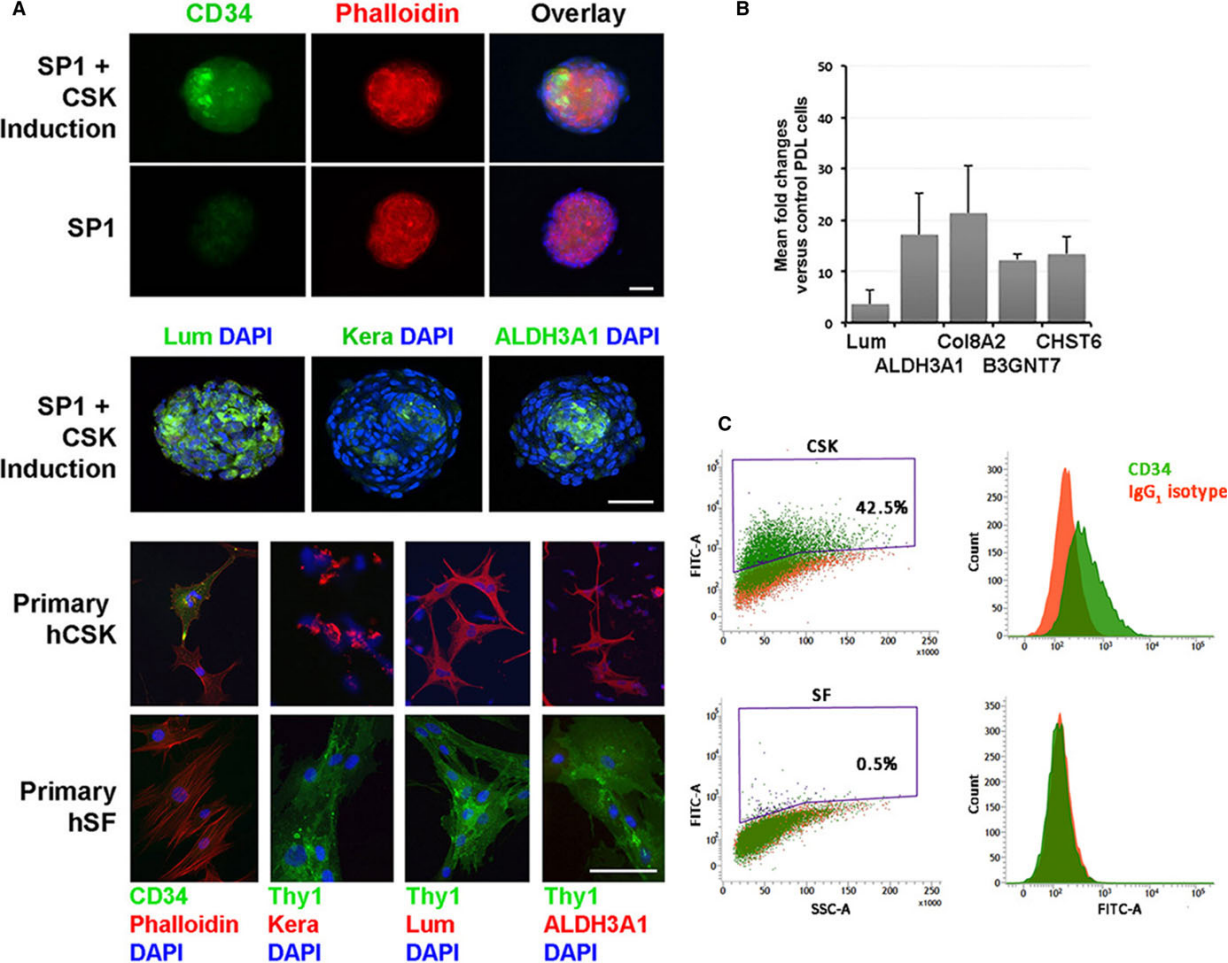
Characterization of periodontal ligament (PDL) spheroids under corneal stromal keratocyte (CSK) differentiation. Spheroids were generated by protocol having 5% chick embryo extract and treated with bFGF, TGFβ3 and _L_A_2_P on low attachment surface for 7 d. A, By confocal microscopy, the expression of CD34, Lum, KERA and ALDH3A1 was visualized in treated spheroids, and this was similarly observed in primary human CSKs but not in stromal fibroblasts (SFs). B, qPCR analysis showing the up‐regulated fold changes of CSK genes (*LUM*,* ALDH3A1*,* Col8A2*,* B3GNT7*,* CHST6*) in treated spheroids compared to control PDL cells. C, Flow cytometric dot plots and histograms showing the event profiling of CD34 expression in human primary CSK (42.5% CD34^+^ cells) and SF (0.5% CD34^+^ cells)

When the spheroids were cultured under CSK induction with bFGF (20 ng/mL), TGFβ3 (1 ng/mL) and _L_A_2_P (1 mmol/L) on low attachment surface for 5‐7 days, they expressed CD34, LUM, ALDH3A1, Col8A2 and low levels of KERA and its synthesizing enzymes (B3GNT7, CHST6) as shown by immunostaining and qPCR analyses (Figure 3A,B). *LUM* up‐regulation was not clear under qPCR, and this could be because of the high basal expression levels. Without induction, these CSK‐associated genes were barely expressed (Figure 3A top panel). In particular, CD34 immunoreactivity was detected in part of the treated spheroids, similar to that observed in primary human CSK (Figure 3A bottom panel). We found almost half of human primary CSK population expressed CD34. Flow cytometry also revealed that 42.5% CSKs were CD34^+^ (Figure 3C). Concomitantly, all human CSKs were positive to LUM, KERA and ALDH3A1 (Figure 3A). However, human SFs did not express any CSK genes and only 0.5% cells were CD34 positive as revealed by flow cytometry (Figure 3A,C).

**Figure 4:**
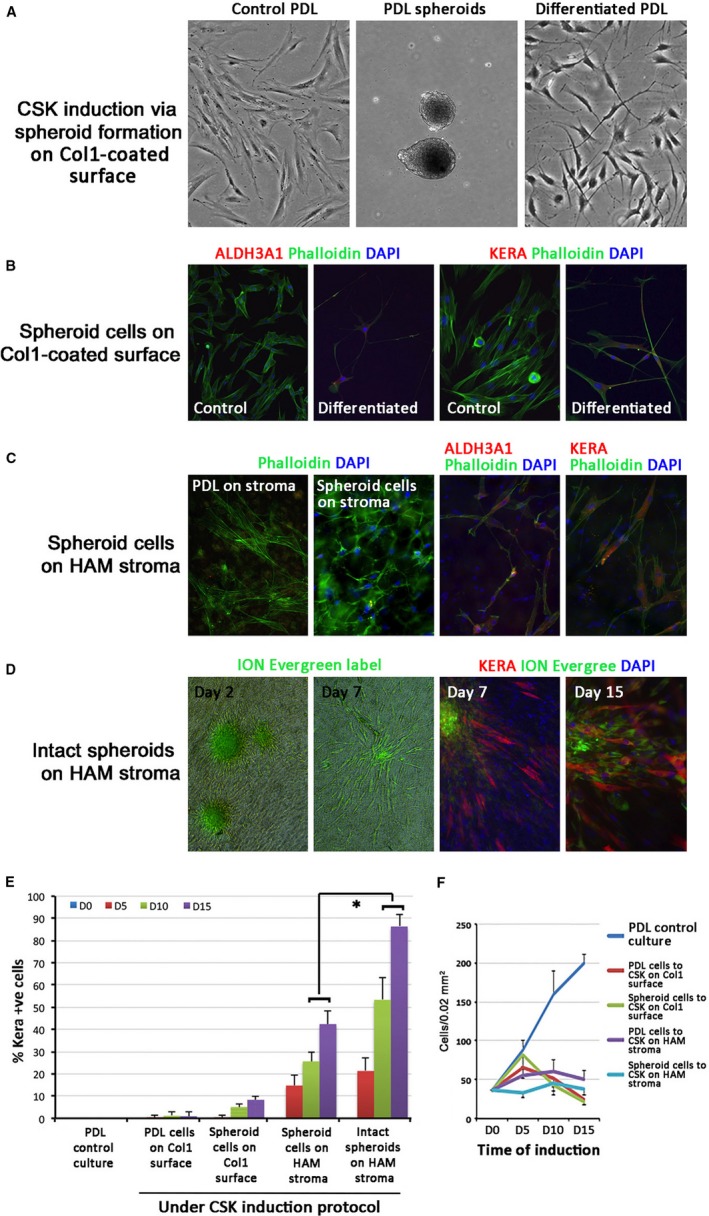
Differentiation of periodontal ligament (PDL) spheroids on amnion stroma to corneal stromal keratocyte (CSK) phenotypes. A, Primary PDL cells generated spheroids and dissociated spheroid cells were induced differentiated to CSK‐like cells with dendritic shape. B, Dissociated spheroid cells showed mildly up‐regulated ALDH3A1 and KERA compared to control PDL cells. C, Dissociated spheroid cells differentiated on human amnion (AM) stroma showed dendritic morphology, compared to PDL cells on AM stroma (without spheroid formation) and expressed ALDH3A1 and KERA. D, Differentiation of intact PDL spheroids on AM stroma. Cell migration from spheroids at day 2 and 7 and KERA expression at day 7 and 15 were compared. E, Percentage of KERA expressing cells at various conditions of differentiation. The greatest efficiency was observed for PDL spheroids differentiated on AM stroma. *P <* *.05, compared at same day of induction; Mann‐Whitney *U*‐test. F, Cells exhibited growth arrest during CSK differentiation

**Figure 5:**
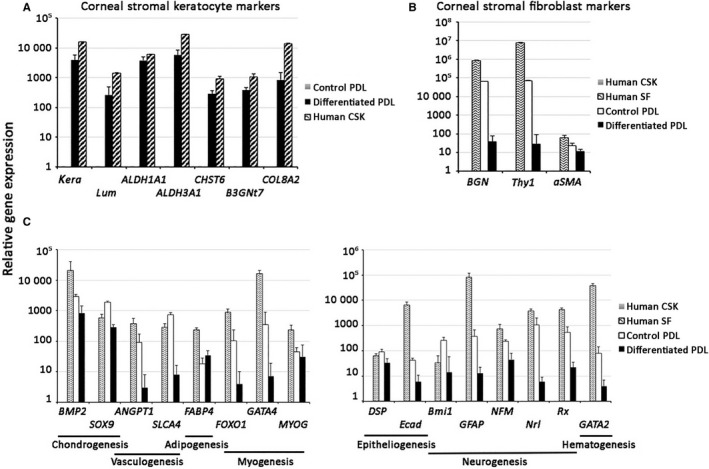
RNA expression study of periodontal ligament (PDL) spheroid cells differentiated on human amnion stroma. Markers characterizing different lineages, including (A) corneal stromal keratocyte; (B) stromal fibroblasts (SFs); (C) others: vasculogenesis, adipogenesis, myogenesis, epitheliogenesis, neurogenesis and hematogenesis, among control and differentiated PDL cells, human corneal stromal keratocyte (CSK) and SF. Reference (set as 1) for gene expression comparison in A: control PDL; in B and C: human CSK

When spheroids were dissociated into single cells using collagenase, followed by induced differentiation to CSK lineage on collagen I‐coated surface, the cells attached and displayed dentritic morphology with thin processes extending and interacting with neighbouring cells (Figure 4A). They had mildly up‐regulated expression of ALDH3A1 and KERA (Figure 4B). When cells were differentiated on AM stromal side, stronger ALDH3A1 and KERA expression was noted while the cells maintained highly dendritic morphology, which was not observed for PDL cells differentiated on AM stroma without spheroid formation (Figure 4C). The percentage of KERA‐expressing cells in five random regions was gradually increased, reaching 42.4 ± 6.15% for spheroid‐dissociated cells on AM stroma, compared to 9.2 ± 0.9% for cells on collagen 1‐coated surface, at day 15 (Figure 4E). CSK induction was significantly improved when intact spheroids were directly seeded on AM stroma (Figure 4D). While cells were migrating from the spheroids into AM stroma, 86.4 ± 5.6% cells had KERA expression after 15 days of treatment (*P *<* *.05, Mann‐Whitney *U*‐test) (Figure 4E). Using qPCR analysis, the differentiated PDL cells had up‐regulated expression of CSK genes (*KERA*,* LUM*,* ALDH1A1*,* ALDH3A1*,* CHST6*,* B3GNT7*,* COL8A2*) (Figure 5A). They also exhibited growth arrest starting at the first 5 days of induction and the number was maintained until 15th day, the longest induction time in our experiment (Figure 4F). This indicated that the differentiated cells were quiescent, mimicking mature CSKs. Of note, the fibroblast genes (*BGN*,* Thy1*, α*SMA*) were clearly suppressed, when compared to human SFs or control PDL cells (Figure 5B). We further studied the marker gene expression related to other differentiation lineages (Figure 5C). In general, the differentiated PDL cells from spheroids cultured on AM stroma exhibited only minor variation for gene expression characteristic of vasculogenesis (*ANGPT1*,* SLCA4*), adipogenesis (*FABP4*), myogenesis (*FOXO1*,* GATA4*,* MYOG*), epitheliogenesis (*DSP*,* Ecad*), neurogenesis (*BMI1*,* GFAP*,* NFM*,* Nrl*,* Rx*) and hematogenesis (*GATA2*), when compared to human primary CSKs. The relatively strong expression of chondrogenesis marker, *BMP2* (837 ± 590 folds) and *SOX9* (283 ± 60 folds), could be related to the residual NC gene expression after spheroid enrichment.

### 3.3 Intrastromal spheroid injection to porcine corneas and organ culture

**Figure 6:**
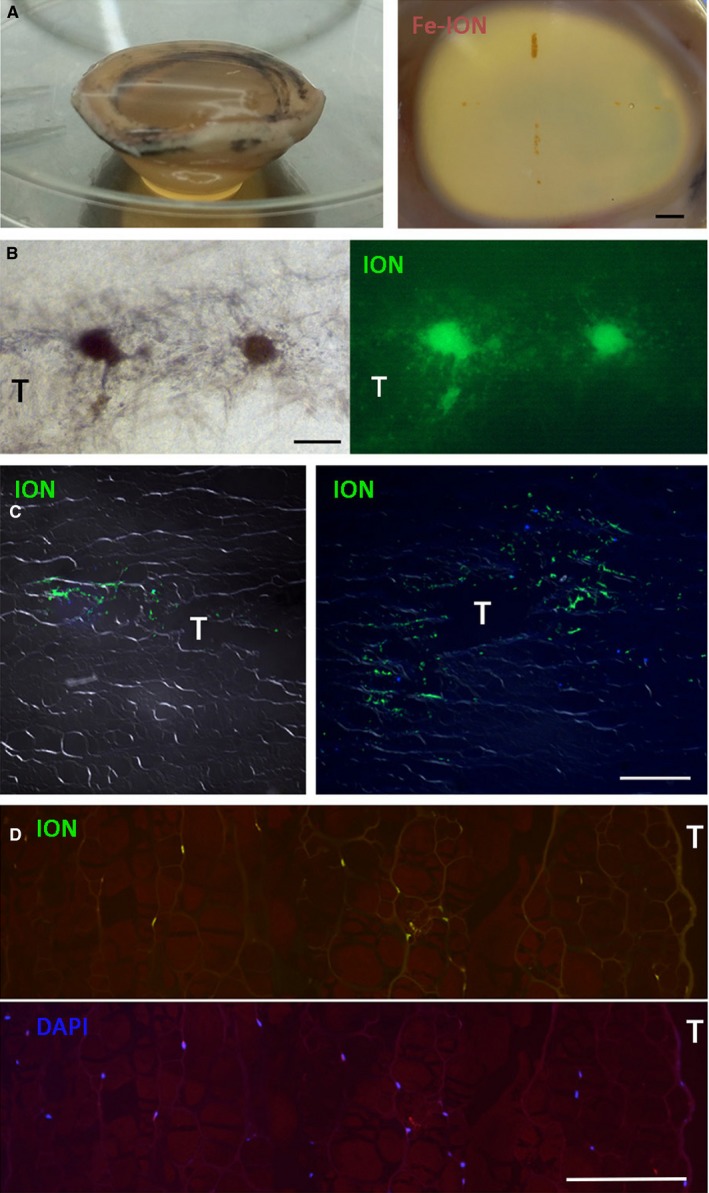
Intrastromal spheroid injection to porcine corneas and organ culture. A, Periodontal ligament (PDL) spheroids labelled with Molday ION‐Evergreen™ were injected into stromal tunnels made on porcine corneas supported by low melting agarose. B, Migration of ION‐Evergreen labelled PDL cells from spheroids to porcine stroma. C, Movement of PDL cells inside porcine stroma at day 2 and 7 post‐injection. D, Extensive migration of PDL cells after 14 d post‐injection. Scale bars: 1 mm (A); 100 μm (B‐D)

We assessed the ability of PDL spheroids to express CSK phenotype after intrastromal injection to porcine corneas. After suspension culture for 5 days, the spheroids (>50 μm diameter) were labelled with Molday ION‐Evergreen™ reagent for 48 hours before collection. The washed spheroids were suspended in normal saline and injected into porcine corneas via stromal tunnels (Figure 6A). Around 10‐20 spheroids were delivered into each tunnel and a total of up to 80 in each cornea. The corneas were placed in organ culture in CSK induction medium for 7‐14 days. At day 2, the ION‐Evergreen labelled cells emitting green fluorescence were visualized to migrate from the spheroids into the host stromal tissue (Figure 6B). Tissue sections revealed additionally a time‐dependent movement of labelled cells from the injection tunnel to the surrounding stroma (Figure 6C). After 14 days, we observed that the cells migrated up to 300 μm distance from the injection site and they were evenly distributed inside the stroma (Figure 6D).

**Figure 7:**
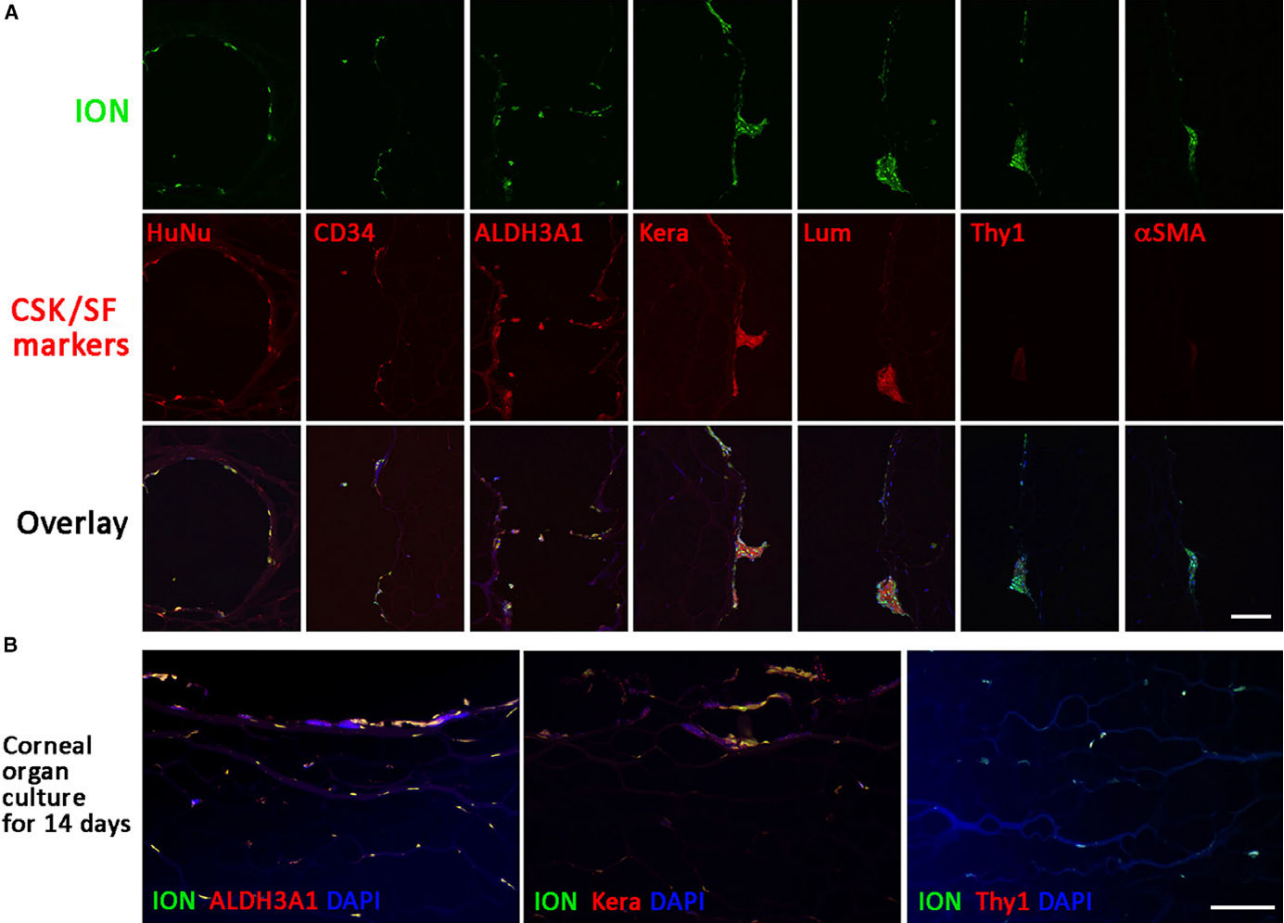
Characterization of periodontal ligament spheroid cells after intrastromal injection to porcine corneas. Immunostaining of corneal stromal keratocyte (CSK) and stromal fibroblast (SF) markers after organ culture for 7 d (A) and 14 d (B). Scale bars: 50 μm

After corneal organ culture for 7 days, immunostaining of corneal sections was performed using antibodies unreactive to porcine antigens and the results were assessed for the co‐localization to the ION Evergreen‐labelled human PDL cells. The exclusive co‐localization of human nuclear (HuNu) antigen with ION labels confirmed the specific visualization of human cells (Figure 7A). The expression of CD34, ALDH3A1, KERA and LUM was detected, indicating that these cells attained CSK characteristics. These differentiated cells had negligible expression of Thy1 and αSMA. At day 14 of culture, much stronger expression of ALDH3A1 and KERA was noted around the injected cells, whereas Thy1 expression was still undetectable (Figure 7B). These data indicated that PDL cells from spheroids were induced to differentiate towards CSK phenotype, and the differentiated cells were maintained inside the corneal stromal environment.

## 4 DISCUSSION

This study showed that human postnatal PDL containing NC‐derived stem cells were able to differentiate and assume CSK phenotype under a two‐step protocol involving spheroid formation followed by growth factor and cytokine induction on a stromal niche (including human amnion stroma and porcine corneal stroma). The five randomly selected primary PDL cultures from healthy non‐smoking donors exhibited similar efficiencies in generating cells expressing CSK markers (CD34, ALDH3A1, KERA and LUM). Introduction of spheroids through stromal tunnels into porcine corneas resulted in migration of PDL‐differentiated CSK‐like cells inside the host stroma after 14‐day organ culture. Their quiescent nature and uniform cell distribution resembled to that of mature CSKs inside the native stroma. Our results thus demonstrated the potential translation of PDL cells for regenerative corneal cell therapy.

The primary PDL cells were expanded in monolayer culture during which they expressed various NC genes (nestin, Snai1, Slug), MSC markers (CD44, 73, 90, 105, 166, SSEA4, STRO‐1), multipotency genes (Nanog, Klf4) and LUM, which have been previously reported.^22^, ^31^, ^35^, ^40^, ^41^, ^42^ In addition, they demonstrated multipotency, such as osteogenesis under induction with dexamethasone, β‐glycerophosphate and ascorbic acid 2‐phosphate. When the monolayer cells were placed in low attachment culture supplemented with CEE, they formed spheroids with an efficiency of 4.8%. This was similar to our earlier report showing ~4% efficiency.^35^ Spheroid formation appeared to be essential for PDL cells to differentiate towards CSK lineage. Previous studies have shown that CSKs existing in pellet or spheres maintain mesenchymal identity as shown by vimentin expression,^43^ and the cells interacted with collagen and proteoglycan‐rich ECM via integrin‐mediated attachment.^44^ We studied two NCSC enrichment protocols for PDL cells and found that the supplementation with CEE resulted in spheroids expressing nestin, Sox2 and Sox10 while the protocol added with 20 ng/mL EGF derived spheroids expressing nestin only. The latter protocol was reported to expand palatal NCSCs^39^; however, it seems not applicable for PDL cells, even though both are originated from cranial NC developmentally.^45^ Hence, further optimization will achieve better protocols to obtain a refined population of NC‐derived stem cells from oral and craniofacial regions. Having enriched NCSCs, these free‐floating spheroids attained CSK phenotype after further treatment with bFGF, TGFβ3 and _L_A_2_P, a reported CSK differentiation protocol.^46^ The spheroid cells positively expressed CD34, ALDH3A1 and Col8A2, markers for CSKs,^47^, ^48^ and they also had low expression levels of KERA and its synthesizing enzymes (B3GNT7, CHST6). In particular, CD34 immunoreactivity was detected in almost half of cell population inside the treated spheroids. This staining pattern was similar to that of primary human CSK culture and flow cytometry analysis further revealed CD34 expression in 42.5% CSKs (Figure 3). CD34 is a transmembrane phosphoglycoprotein with L‐selectin (CD62L) as its most commonly described ligand.^49^ Ubiquitous CD34 expression is related to hematopoietic cells, and it serves as a marker to enrich hematopoietic stem cells for bone marrow transplants.^50^ CD34^+^ non‐hematopoietic lineage cells also represent a distinct subset of cells with enhanced progenitor activity, such as muscle satellite cells, interstitial cells, epithelial progenitors and CSKs.^51^ The CD34^+^ expressing cells could thus represent the quiescent CSKs or stromal progenitors,^52^ whereas CD34^−^ cells are the activated keratocytes not yet fully reverted to the quiescent condition.^53^ The possibility of fibroblast transition is excluded by the absence of Thy1 expression.^37^, ^38^ We attempted to sort for CD34^+^ cells after spheroid dissociation using various enzymes (including collagenase I, III, TrypLE Select, Accutase and trypsin‐EDTA); however, CD34 was no longer detectable in any of these single cells, indicating that the cell surface CD34 epitope was destroyed by enzyme treatment (data not shown).

The ability of PDL cells to differentiate to CSK appears to require an environment that allows the formation of a three‐dimensional cell synctium. Stromal niche with its ECM component contains a complex network of macromolecules, including proteins, glycoproteins, proteoglycans and polysaccharides with different physical and biochemical properties.^54^ The dynamic and interactive cell‐ECM biology contributes to cell survival, proliferation and differentiation.^55^, ^56^ In our study, when spheroid‐dissociated cells were directly seeded to human AM stroma and cultured under CSK induction protocol for 15 days, much stronger expression of CSK markers (ALDH3A1, KERA) was noted and the cells maintained dendritic morphology, resembling that of CSKs. The percentage of KERA‐expressing cells, regarded as an indicator of CSK induction efficiency, was significantly increased to 42%, when compared to 9% for PDL cells treated on collagen I‐coated surface. This efficiency was further enhanced to 86% when the spheroids were directly differentiated on AM stroma and KERA‐expressing cells migrated extensively into the stromal tissue. This suggests that the AM stromal niche may confer some important physical, biochemical and/or biomechanical properties that are essential to regulate CSK lineage differentiation of PDL cells.^57^, ^58^

Owing to the size range (50‐100 μm diameter), these spheroids may not be easily injected into mouse and rabbit corneas. Instead, thicker corneas, such as in porcine, dog and non‐human primate, are more appropriate models as they are similar to human. Our results showed that PDL cells maintained CSK phenotype while they gradually spread throughout the porcine stroma during organ culture. This demonstrated the 3D arrangement of the differentiated cells and the cell alignment pattern resembled that of native porcine stroma. Further spheroid injection experiments on living species with similar corneal thickness to humans would be required to demonstrate the long‐term cell viability, maintenance of CSK phenotype by the differentiated cells, effect on corneal transparency, stromal architecture as well as host immunity.

Several cell types have been reported for potential use in regenerative corneal therapy.^59^ The recent success in propagating primary human activated keratocytes and the reversion to quiescenct CSKs has “in principal” resolved the issue of CSK shortage which has been a challenging topic in corneal cell therapy.^37^ However, the slow growth ability and the tendency towards a fibroblastic phenotype under prolonged culture are still the limiting factors restricting its therapeutic potential. Also, the issue of long‐term CSK maintenance in wounded cornea conditions and the need of multiple injection to improve the therapeutic efficiency are the prime questions to be solved prior to the translational application. Corneal fibroblasts have been assessed for the regenerative cell therapy,^60^, ^61^ but they do not have any therapeutic benefit because of the negligible production of KSPGs that is important to maintain corneal transparency as well as the production of repair‐type ECM proteins, like fibronectin and proteases, that are involved in stromal remodelling. These cells can further transform into highly contractile myofibroblasts (under the action by TGFβ and platelet‐derived growth factor) that are shown to associate with corneal haze development and scar formation.^62^, ^63^ Human corneal stromal stem cells (CSSCs) identified from limbal stroma have offered an opportunity to develop functional CSKs through population doublings and produce stroma‐like ECM.^64^ Future identification of unique markers will help in a more efficient isolation of pure CSSCs and a better control of differentiation.^65^, ^66^ The report of in vitro differentiating human ESC into CSK via NC progenitors has supplied valuable information for protocol design^46^; however, a number of limitations, such as induction efficiency, control of differentiated cell purity and the risk of tumorigenesis, remain to be solved before its translational application. Adult bone marrow‐ and adipose‐derived MSCs has been shown to differentiate towards CSK phenotype when intrastromally injected into mouse corneas,^67^, ^68^, ^69^ but it is difficult to control the final cell fate. Dental stem cells have been an attractive option in cell regeneration research. The harvest of PDL cells and DPSCs from extracted teeth is a significant advantage when compared to bone marrow, umbilical cord and adipose‐derived MSCs, which require more invasive procedures. Dental SCs are also present in all teeth, of which the third molar is a common source, because of its large tooth volume and surface area. It is the last tooth to develop and is often impacted and remains buried in an “unused,” but healthy state. Third molar extraction is one of the most commonly performed procedures in oral surgery. Impacted third molars are removed to prevent risks of caries, periodontal disease, pericoronitis, odontogenic cysts and dental crowding. Extracted teeth are merely disposed as medical waste.^70^ It is thus under minimal ethical concerns to utilize these cells and future translational application with donor matching or autogenic usage can be supported through “dental cell banking” concept.^19^

Our study highlighted the plasticity and differential capacity of PDL cells to generate CSKs. These cells are readily propagated ex vivo, hence offering an ample amount of viable cells for CSK differentiation for regenerative corneal cell therapy and stromal tissue engineering applications in the management of corneal opacities and corneal biomechanical pathologies.

## CONFLICT OF INTEREST

No conflicting relationship exists for any author.

## AUTHOR CONTRIBUTIONS

Yam and Mehta designed the study; Yam, Teo, Setiawan, Lovatt and Yusoff performed experiments and data acquisition; Yam, Fuest and Mehta interpreted data; Goh collected teeth; and Yam and Mehta prepared manuscript.
