# Supplementary material for: Postnatal periodontal ligament as a novel adult stem cell source for regenerative corneal cell therapy
Source: J Cell Mol Med. 2018 Mar 13;22(6):3119–32. doi: 10.1111/jcmm.13589 (PMC5980160; doi:10.1111/jcmm.13589)
Supplement: Supplementary file 1 [file JCMM-22-3119-s001.docx]

**Induced differentiation of human periodontal ligament-derived stem cells into cells expressing corneal keratocyte phenotype**

Gary Hin-Fai Yam, Ericia Pei-Wen Teo, Melina Setiawan, Matthew J Lovatt, Nur Zahirah Binte M Yusoff, Matthias Fuest, Bee-Tin Goh, Jodhbir S Mehta

**Supporting information**

**Supplementary Table 1.** Donor cornea information

| **Donor** | **Age** | **Gender** | **Cause of death** | **Preservation in Optisol before cell culture (days)** |
| --- | --- | --- | --- | --- |
| 1 | 50 | M | Hepatocellular cancer | 6 |
| 2 | 35 | M | Aspiration pneumonia | 8 |
| 3 | 64 | F | Heart failure | 8 |

F: female; M: male

**Supplementary Table 2:** Antibodies used in this study

|  | **Antibody [clone]** | **Source** | **Applications in this study:**  **working concentration** |
| --- | --- | --- | --- |
| 1 | CD14 [134620] | R&D Systems | Flow cytometry: 0.2 μg/10^6^ cells |
| 2 | CD31-FITC [WM-59] | eBioscience | Flow cytometry: 0.2 μg/10^6^ cells |
| 3 | CD34 [QBEND/10] | Millipore | Immunostaining: 0.2 μg/ml |
| 4 | CD34-APC | BD Biosciences | Flow cytometry: 0.5 μg/10^6^ cells |
| 5 | CD44 [2F10] | R&D Systems | Flow cytometry: 0.2 μg/10^6^ cells |
| 6 | CD45 [2D1] | R&D Systems | Flow cytometry: 0.2 μg/10^6^ cells |
| 7 | CD73 [4G4] | BD Biosciences | Flow cytometry: 0.2 μg/10^6^ cells |
| 8 | CD90/Thy1 | BD Biosciences | Flow cytometry: 0.2 μg/10^6^ cells |
| 9 | CD105 | R&D Systems | Flow cytometry: 0.2 μg/10^6^ cells |
| 10 | CD166 | R&D Systems | Flow cytometry: 0.2 μg/10^6^ cells |
| 11 | Keratocan, KERA | Sigma | Immunostaining: 1:100 |
| 12 | Lumican, LUM | Sigma | Immunostaining: 1:100 |
| 13 | Aldehyde dehydrogenase 3A1, ALDH3A1 | Proteintech | Immunostaining: 1:100 |
| 14 | α-Smooth muscle actin (αSMA) [1A4] | DAKO | Immunostaining: 1:100 |
| 15 | Nestin [10c2] | SantaCruz | Immunostaining: 1:150 |
| 16 | Sox2 [H65] | SantaCruz | Immunostaining: 1:100 |
| 17 | Sox10 [H2] | SantaCruz | Immunostaining: 1:100 |
| 18 | Connexin43 [4E6.2] | Millipore | Immunostaining: 1:100 |
| 19 | STRO-1 | Invitrogen | Immunostaining: 1:100 |
| 20 | SSEA4 [MC813-70] | Millipore | Immunostaining: 1:100 |
| 21 | Phalloidin-Alexa568 | Invitrogen | Immunostaining: 1:500 |
| 22 | Human Nuclei (HuNu) [235-1] | Millipore | Immunostaining: 1:200 |
| 23 | Isotype-specific IgG | BD Biosciences | Flow cytometry: 0.2 μg/10^6^ cells |
| 24 | AlexaFluor 488 goat anti-mouse/rabbit IgG (H+L) | Jackson ImmunoRes Lab | Immunostaining: 1:700  Flow cytometry: 1:500 |
| 25 | RedX-conjugated goat anti-mouse/rabbit IgG (H+L) | Jackson ImmunoRes Lab | Immunostaining: 1:700  Flow cytometry: 1:500 |

**Supplementary Table 3.** Expression primers for qPCR study

|  | **Gene** | **GeneBank Accession No.** | **Sequences (5’-3’)** | **Product size (bp)** |
| --- | --- | --- | --- | --- |
| 1 | ALDH1A1 | NM_000689.4 | F: CTGGTTATGGGCCTACAGCA  R: ATTGTCCAAGTCGGCATCAG | 191 |
| 2 | ALDH3A1 | NM_001135168.1 | F: CATTGGCACCTGGAACTACC  R: GGCTTGAGGACCACTGAGTT | 87 |
| 3 | ANGPT1 | NM_001199859.1 | F: TGGGGGAGGTTGGACTGTAA  R: TGCCTCTGACTGGTAATGGC | 150 |
| 4 | B3GNT7 | NM_145236.2 | F: AGTCTCACCCCTGGTCAGTT  R: AGCAGTTAGTGGTGGTCACG | 187 |
| 5 | BGN | NM_001711.4 | F: GAGGACCTGCTTCGCTACTC  R: CCCTGGCCAACTTGTTGTTG | 136 |
| 6 | Bmi1 | NM_005180.8 | F: TCATCCTTCTGCTGATGCTG  R: GCATCACAGTCATTGCTGC | 221 |
| 7 | BMP2 | NM_001200.2 | F: ACTCGAAATTCCCCGTGACC  R: CCACTTCCACCACGAATCCA | 144 |
| 8 | CHST6 | NM_021615.4 | F: TACCGGCCTGTGTACTCTGA  R: ACTAATTTCGGGGGTGCGAG | 118 |
| 9 | Col8A2 | NM_005202.2 | F: ACATCCAGCCCATGCAGAAA  R: GCATTTCCAGGTACTGGCCT | 72 |
| 10 | DSP | NM_004415.2 | F: CAAAGCCGACCTGCGCGAGA  R: GTCCACGGCCAGTGGGTTCG | 150 |
| 11 | FABP4 | NM_001442.2 | F: TGGCATGGCCAAACCTAACA  R: TCCTGGCCCAGTATGAAGGA | 108 |
| 12 | FOXO1 | NM_002015.3 | F: AGTGGATGGTCAAGAGCGTG  R: CCCCAGGATCAACTGGTGTC | 108 |
| 13 | GAPDH | NM_002046 | F: TGTGGTCATGAGTCCTTCCA  R: CGAGATCCCTCCAAAATCAA | 294 |
| 14 | GATA2 | NM_032638.4 | F: CAGACGACAACCACCACCTTATG  R: TGGTCAGTGGCCTGTTAACATTG | 106 |
| 15 | GATA4 | NM_002052.3 | F: AAGCCCAAGAACCTGAATAAATC  R: TGGCGTTGCTGGAGTTG | 100 |
| 16 | GFAP | NM_002055.4 | F: ACCAGGACCTGCTCAATGTC  R: ATCTCCACGGTCTTCACCAC | 200 |
| 17 | Kera | NM_07035.3 | F: ATCTGCAGCACCTTCACCTT  R: CATTGGAATTGGTGGTTTGA | 167 |
| 18 | Lum | NM_002345.3 | F: CCTGGTTGAGCTGGATCTGT  R: TGGTTTCTGAGATGCGATTG | 194 |
| 19 | MYOG | NM_002479.5 | F: GATCATCTGCTCACGGCTGA  R: GGTTTCATCTGGGAAGGCCA | 111 |
| 20 | Nestin | NM_006617.1 | F: CAGGAGAAACAGGGCCTACAGA  R: TCCAGCTTGGGGTCCTGAA | 191 |
| 21 | NFM | NM_005382.2 | F: AGCCTTGACTTCAGCCAGTC  R: TGAGCCTTCTCGTGGTTCAC | 190 |
| 22 | Nrl | NM_006177.2 | F: GGCTCCACACCTTACAGCTC  R: CTGGGCTCCCTGGGTAGTAG | 219 |
| 23 | p75^NTR^ | NM_002507.3 | F: TCATCCCTGTCTATTGCTCCA  R: TGTTCTGCTTGCAGCTGTTC | 93 |
| 24 | PCNA | NM_002592.2 | F: ACGTCTCTTTGGTGCAGCTCA  R: CATTGCCGGCGCATTTTA | 123 |
| 25 | Rx | NM_013435.2 | F: AGCGAAACTGTCAGAGGAGGAA  R: TCATGCAGCTGGTACGTGGTGAA | 81 |
| 26 | Slug | NM_003068.4 | F: TTCGGACCCACACATTACCT  R: GCAGTGAGGGCAAGAAAAAG | 122 |
| 27 | Snai1 | NM_005985.3 | F: GACCCCAATCGGAAGCCTAACTA  R: AGCCTTTCCCACTGTCCTCATCT | 164 |
| 28 | SOX9 | NM_000346.3 | F: TTCACCTACATGAACCCCGC  R: CAAGGTCGAGTGAGCTGTGT | 134 |
| 29 | Thy1 | NM_006288 | F: GACCCGTGAGACAAAGAAGC  R: TGGAGTGCACACGTGTAGG | 159 |

**Supplementary Table 4. Percentage of PDL cells expressing different CD markers by flow cytometry**

| **Cells** | **CD14** | **CD31** | **CD34** | **CD44** | **CD45** | **CD73** | **CD90** | **CD105** | **CD166** |
| --- | --- | --- | --- | --- | --- | --- | --- | --- | --- |
| hPDL13 | 3% | 0.5% | 0.5% | 96% | 33% | 97% | 93% | 96% | 98% |
| hPDL15 | 15% | 4% | 4% | 97% | 71% | 90% | 97% | 99% | 99% |
| hPDL27 | 22% | 1% | 0.3% | 99% | 0.7% | 99% | 99% | 99% | 99% |
| hPDL34 | 12% | 10% | 0.5% | 99% | 64% | 99% | 97% | 99% | 99% |
| hPDL43 | 35% | 7% | 3% | 99% | 48% | 99% | 99% | 99% | 99% |

**
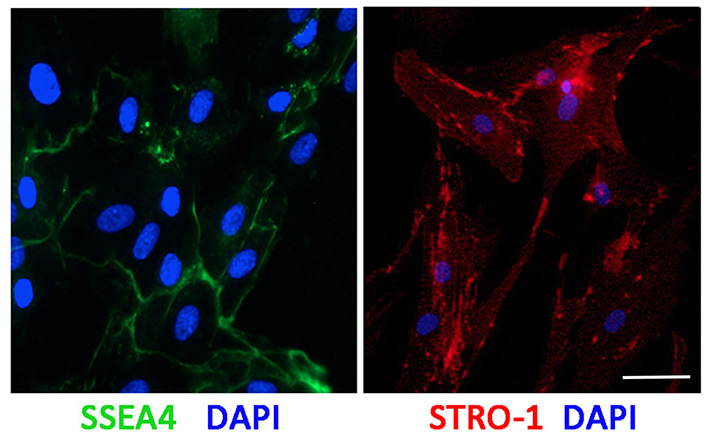
**

**Supplementary Figure 1.** Representative immunofluorescence images showing the positive expression of SSEA4 and STRO-1 in primary human PDL cells. Both were expressed predominantly on the cell surface and partially in the cytoplasm. Scale bar: 50 μm.


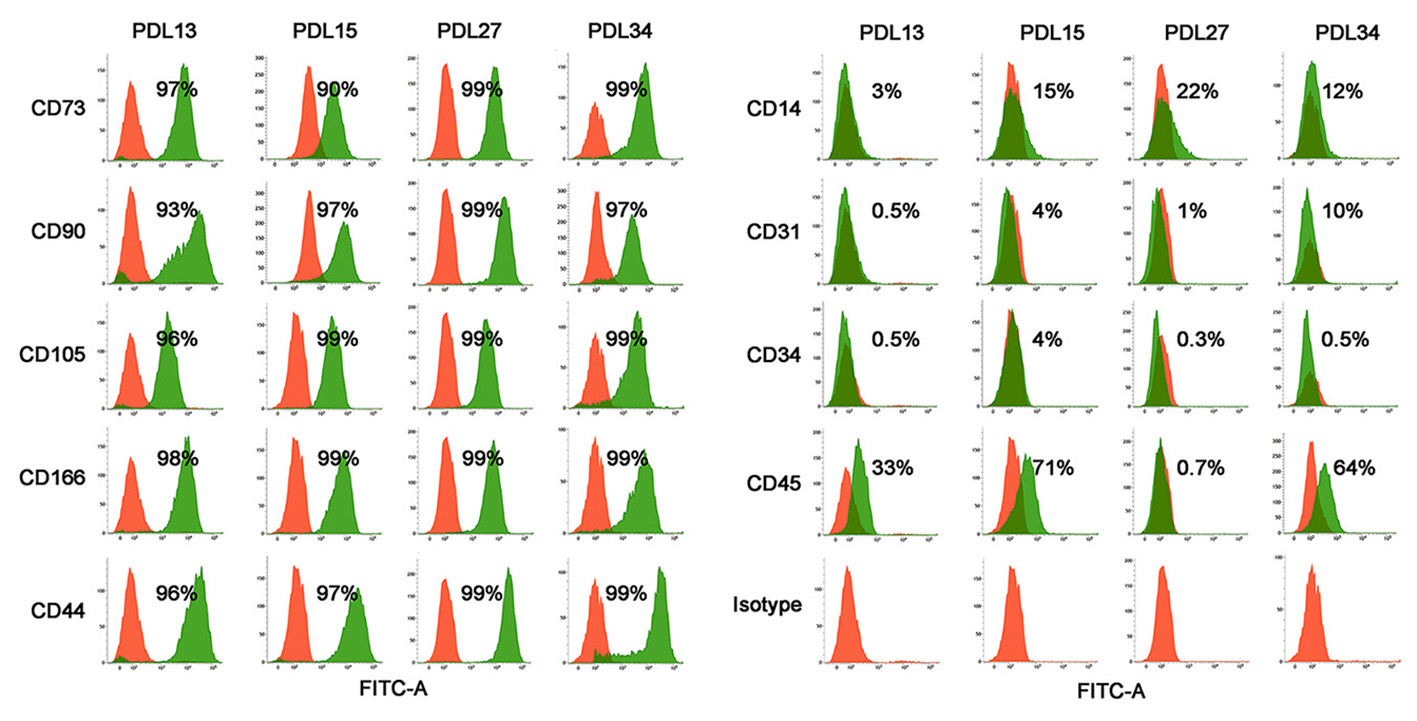


**Supplementary Figure 2.** Flow cytometric histograms showing the event profiling of CD markers for mesenchymal stem cells, monocytes, leukocytes and hematopoietic lineage cells in different primary human PDL cells. Red-colored histograms denote the isotype control.


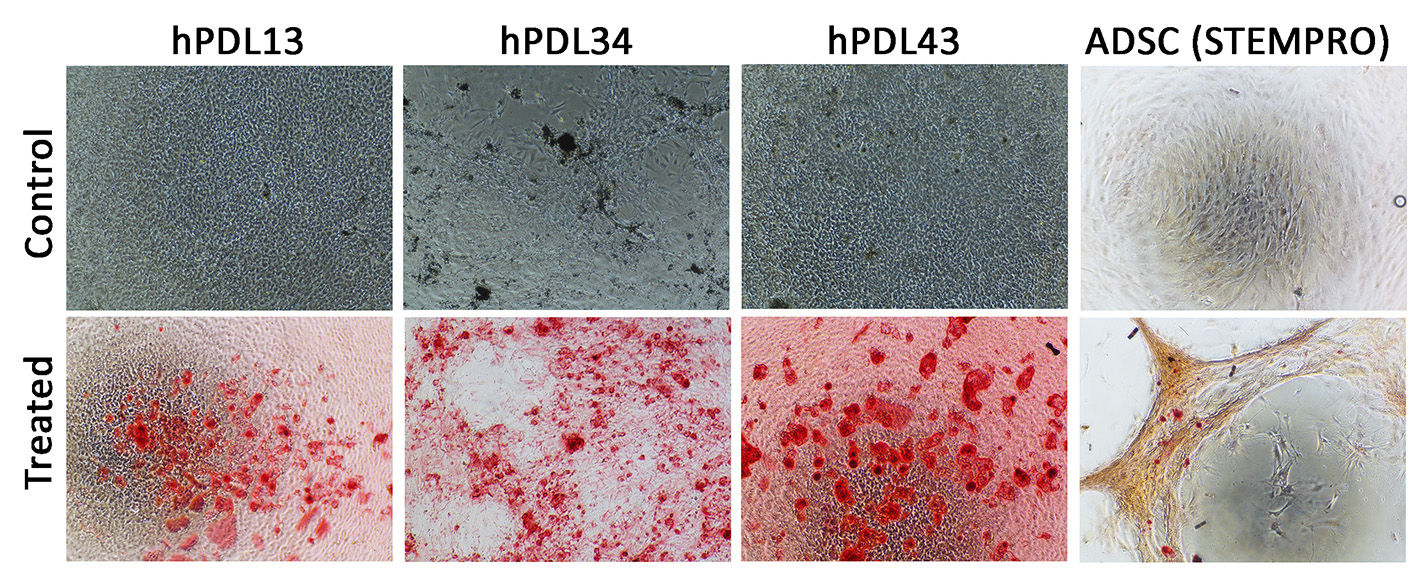


**Supplementary Figure 3.** Representative phase contrast images showing the induced differentiation of 3 primary human PDL cells towards osteogenesis as documented by lineage specific staining of Alizarin Red S. Human adipose-derived MSCs (ADSCs) were used in parallel control experiment.

Method: Human primary PDL cells at P3 to 4 and human ADSCs (STEMPRO, ThermoFisher Scientific, US) were seeded at a density of 104 cells/cm2 in MesenPRO RS^TM^ medium (ThermoFisher Scientific) containing 2% FBS. At 70% confluence, the cells were treated with STEMPRO Osteogenesis kit (ThermoFisher Scientific) for 21 days with fresh medium replenished every 2 days. The cells were fixed with neutral buffered 3% paraformaldehyde (Sigma-Aldrich), stained with Alizarin Red S (Sigma-Aldrich) and visualized under phase contrast microscopy.
